# Use of Cepheid Xpert Carba-R® for Rapid Detection of Carbapenemase-Producing Bacteria in Abdominal Septic Patients Admitted to Intensive Care Unit

**DOI:** 10.1371/journal.pone.0160643

**Published:** 2016-08-04

**Authors:** Andrea Cortegiani, Vincenzo Russotto, Giorgio Graziano, Daniela Geraci, Laura Saporito, Gianfranco Cocorullo, Santi Maurizio Raineri, Caterina Mammina, Antonino Giarratano

**Affiliations:** 1 Department of Biopathology and Medical Biotechnologies (DIBIMED), Section of Anesthesia, Analgesia, Intensive Care and Emergency, Policlinico P. Giaccone, University of Palermo, Via del Vespro 129, 90127, Palermo, Italy; 2 Department of Sciences for Health Promotion and Mother-Child Care, University of Palermo, Palermo, Italy; 3 Department of General Surgery, Urgency and Organ Transplantation, University of Palermo, Italy; Wadsworth Center, UNITED STATES

## Abstract

Early institution of effective antibiotic therapy and source control are pivotal to improve survival of abdominal septic patients. Xpert^®^ Carba-R is a real time polymerase chain reaction assay for rapid detection and differentiation of five genes (*bla*_*KPC*_, *bla*_*VIM*_, *bla*_*OXA-48*_, *bla*_*IMP-1*_, *bla*_*NDM*_) responsible for carbapenem resistance. We performed an observational study investigating the clinical usefulness and applicability of Xpert^®^ Carba-R to detect carbapenem resistance in abdominal septic patients admitted to intensive care unit. We compared the results of Xpert^®^ Carba-R with standard microbiological culture. We collected a set of two rectal/stomia swabs and two swabs from abdominal drainage fluid for each patient. We included 20 patients for a total of 45 comparisons between the two methods. In our clinical setting, the overall performance of Xpert® Carba-R for detection of carbapenem resistance in the presence of genes detectable and non-detectable by the method was: sensitivity 50% (95% CI 24.6–75.3); specificity 93.1% (95% CI 77.2–99.1); positive predictive value (PPV) 80% (95% CI 44.4–97.5); negative predictive value (NPV) 77.1% (95% CI 56.9–89.6). The inter-rater agreement was 0.47 (SE 0.14; 95% CI 0.20–0.74). When considering the only 5 mechanisms of resistance detected by both methods, the overall diagnostic performance was: sensitivity 100% (95% CI 69.1–100), specificity 94.2 (95% CI 80.8–99.3), PPV 83.3 (95% CI 59.6–97.9) and NPV 100% (95% CI 89.4–100). The inter-rater agreement was 0.88 (SE 0.08; 95% CI 0.71–1). Xpert® Carba-R may be considered an additional diagnostic tool for early diagnosis of carbapenem resistance in abdominal septic patients. Clinicians should be aware of their epidemiology before its introduction in the diagnostic protocol of their intensive care units.

## Introduction

Infections caused by microorganisms with resistance to carbapenems are associated with a high morbidity and mortality. These microorganisms are frequently responsible for intra-abdominal infections due to the increasing patients’ colonization by multidrug resistant organisms (MDROs). [1–4] Effective antibiotic treatment is a mainstay of management of sepsis of intra-abdominal origin, in association with early resuscitation and source control. [5–8] Carbapenem resistance makes empirical and targeted treatment of abdominal infections challenging, due to the high risk of ineffectiveness of commonly used antibiotics (e.g. cephalosporins, carbapenem) and the need to adopt alternative antibiotic strategies (e.g. combination regimens). [4, 9] Early detection of antibiotic resistance may be useful for rapid institution of effective antibiotic treatment and for improving patients’ outcome. [7] Conversely, turnaround time needed for culture-based detection of carbapenem resistance is relatively long. [10] Non culture-based methods may reduce the time needed to get the antibiotic resistance data leading to an earlier institution of an effective antimicrobial treatment. [11] For this purpose, polymerase chain reaction (PCR) assays are the most widely used and studied methods. However, their diagnostic performance, agreement with culture-based techniques and clinical usefulness is not definitely established. [11, 12]

Xpert^®^ Carba-R is a real time PCR assay for rapid detection and differentiation of five genes (*bla*_*KPC*_, *bla*_*VIM*_, *bla*_*OXA-48*_, *bla*_*IMP-1*_, *bla*_*NDM*_) associated with non-susceptibility to carbapenem in Gram-negative bacteria, mostly belonging to the *Enterobacteriaceae* family. [1] The reports of its clinical application have been rare and mostly related to the identification of colonization for surveillance and screening purposes. [13, 14] Our aim was to test applicability and reliability of Xpert^®^ Carba-R in a clinical setting characterised by a relatively high prevalence of colonization and infections by carbapenem resistant bacteria. We evaluated the clinical usefulness of Xpert^®^ Carba-R for rapid detection of carbapenem resistant bacteria compared to microbiological culture in critically ill patients with abdominal sepsis.

## Methods

### Study design

We performed a single-centre observational prospective study from September 2014 to December 2015 in the General Intensive Care Unit in cooperation with the Hygiene Section, Department of Science for Health Promotion and Mother-Child Care of the University hospital Policlinico “P. Giaccone”, Palermo, Italy. The Ethics Review Board (Comitato Etico Palermo 1) approved the study protocol and we obtained written informed consent from every included patient or their relatives. The enrolled patients met the following inclusion criteria: 1) severe sepsis or septic shock at the moment of ICU admission or at any time during ICU stay basing on international sepsis criteria [15] by attending physicians; 2) admission to ICU related to either elective or emergent abdominal surgery; 3) proven or suspected intra-abdominal infection as primary focus of septic state. Exclusion criteria were: 1) no informed consent; 2) abdominal surgery not involving the digestive tract, including pancreas and biliary tract; 3) samples for one of the tests obtained more than 30-minutes apart; 4) inability to perform the complete sequence of actions from sampling to processing of the specimens set within 12 hours; 5) readmission within 5 days from the ICU discharge. For every consecutive patient matching the inclusion criteria we obtained a set of two swabs from both rectum or stomia (if any) faecal material and two swabs from fluid of abdominal drainages most close to the site of abdominal surgery. For the purpose of the study, we used the term stomia to indicate any surgical opening of the gastrointestinal tract to the abdominal wall.

One swab from rectum/stomia and one swab from abdominal drainage fluid was sent to the laboratory and processed for standard microbiological cultures. Another swab from rectum/stomia and another from abdominal drainage fluid were tested by Xpert^®^ Carba-R. We performed blood cultures at admission in all included patients according to our institutional protocol. Nurses were in charge for the first part of our ICU protocol aimed to obtain samples from rectum/stomia faecal material and fluid from abdominal drainages for microbiological culture and Xpert^®^ Carba-R.

We registered demographic and clinical data about the enrolled patients including patient origin (e.g. emergency department, surgical ward, operating room), information about abdominal surgery (type, site, urgency, whether first or re-intervention), previous antibiotic regimen, Sequential Organ Failure Assessment score (SOFA) at the time of study inclusion. We also collected the results of microbiological cultures, identified microrganisms, Minimum Inhibitory Concentration (MIC) of imipenem and meropenem, genetic determinants of carbapenem resistance and turn-around time to obtain results of antibiogram. We further recorded results from Xpert^®^ Carba-R in terms of detection of resistance-associated genes, and turn-around time to the result.

### Microbiological cultures and Xpert^®^ Carba-R tests

#### Culture-based methods

For cultural screening each swab, after an overnight incubation in Brain Heart Infusion broth (Oxoid, Basingstoke Hampshire, United Kingdom), was inoculated onto a MacConkey agar plate (Oxoid, Basingstoke Hampshire, United Kingdom) with a 10μg meropenem disk (Oxoid, Basingstoke Hampshire, United Kingdom). MacConkey agar plates were incubated at 35°C overnight. All the morphologically different colonies growing into the meropenem disk halo were picked up and subcultured for purity. The bacterial identification was performed by biochemical standard assays. MIC values of meropenem and imipenem were assessed by E-test strips (BioMerieux, Marcy-l’Etoile, France). The isolates were identified as resistant to carbapenems according to the European Committee on Antimicrobial Susceptibility Testing (EUCAST) breakpoints. [16, 17] We adopted updated EUCAST breakpoints tables (version 4.0 for 2014 and then 5.0 for 2015).

#### PCR-based methods

Molecular analysis was carried out on the swabs using the Cepheid Xpert^®^ Carba-R assay and the GeneXpert^®^ device (Cepheid, Sunnyvale, USA). This test, based on an automated real-time

PCR, is designed for rapid detection and differentiation of the *bla*_KPC_, *bla*_NDM_, *bla*_VIM_, *bla*_OXA-48_, and *bla*_IMP-1_ gene sequences associated with carbapenem-non-susceptibility in Gram-negative bacteria, mostly belonging to the *Enterobacteriaceae* family. All swabs from both rectum/stomia and abdominal drainage fluid were processed according to the same procedure. More in details, each swab was placed into a vial of the sample reagent (Cepheid, Sunnyvale, USA) and vortexed at high speed for 10 seconds. Then, 1.7 ml of the suspension was transferred into the sample chamber of the Xpert^®^ Carba-R cartridge. The results were interpreted by the GeneXpert System from measured fluorescent signals and shown on the View Results window. When carbapenem resistance was detected by microbiological culture, we performed specific in-house PCR for the *bla*_KPC_, *bla*_NDM_, *bla*_VIM_, *bla*_OXA-48_, and *bla*_IMP-1_ [18–21].

Further molecular tests were performed with specific in-house PCR for additional carbapenem resistance genes, namely *bla*_OXA-23_, *bla*_GES_, *bla*_PER_ and *bla*_VEB_, frequently encountered in our setting [18–21], when 1) Xpert^®^ Carba-R resulted negative but microbiological culture showed carbapenem resistance 2) when there was evidence of polymicrobial growth from microbiological culture.

### Statistical analysis

We analysed variables distribution by the D’Agostino-Pearson test. We calculated and reported mean and standard deviation for variables with normal distribution. We expressed variables without normal distribution with median and interquartile range (25^th^-75^th^). We compared variables with normal distribution using the Student’s t test. Mann-Whitney test was used for variables with non normal distribution. We calculated inter-rater agreement using the non-weighted Cohen’s k along with standard error (SE) and 95% confidence interval (95% CI). We interpreted Cohen’s k values according to Altman et al. We considered concordance either the detection of carbapenem resistance by both culture and Xpert Carba-R or the negative results by both methods. We calculated sensitivity, specificity, positive predictive value (PPV) and negative predictive value (NPV) of detecting a carbapenem resistance by Xpert^®^ Carba R when compared to microbiological culture. Our cohort represented a convenience sample of patients with the pre-specified inclusion criteria. We considered a p-value < 0.05 as statistically significant. We used MedCalc^®^ for Windows version 9.5.0.0 (MedCalc Software, Mariakerke, Belgium) for statistical analysis.

## Results

We screened for inclusion a total amount of 49 patients. Eight patients were excluded for lack of informed consent, 16 for the inability to perform the complete sequence of actions (from sampling to processing of the set of specimens) within the pre-specified cut-off time and 5 were excluded because of readmission within 5 days. We included 20 patients in the study. Three patients were included twice in the study since they were readmitted to the ICU after 5 days due to abdominal post surgical complications. We collected a total of 22 complete sets of specimens and one incomplete set since one patient did not have abdominal post-surgical drainage. Characteristics of included patients are summarized in Table 1. Of 23 sets of specimens from either rectum or stomia, 12 were obtained from rectum and 11 from post-surgical stomia. The overall number of comparison between the two methods regardless of the sampling site was 45 (22 complete comparisons + 1 comparison from stomia only). Mean turnaround time for results by Xpert^®^ Carba-R was 88.2 minutes (SD: 8.56) compared to approximately to 72 hours needed for identification of carbapenem resistant strains by culture. Nine out of 23 (39.1%) specimens from rectum/stomia resulted positive for carbapenem resistant bacteria whereas 14 (60.9%) resulted negative by microbiological cultures. Five out of 23 (21.7%) specimens from rectum/stomia resulted positive and 18 (78.3%) resulted negative for Xpert^®^ Carba-R. Concerning specimens from rectum/stomia, 4 resulted positive and 13 resulted negative for both cultural and molecular assays. Five specimens resulted positive for microbiological culture and negative for Xpert^®^ Carba-R. One specimen resulted negative for microbiological cultures and positive for Xpert^®^ Carba-R. Microbiologial cultures from rectum/stomia specimens isolated the following microorganisms: *A*. *baumannii* (5 isolations), *K*. *pneumoniae* (4 isolations), *P*. *aeruginosa* (3 isolations), *Enterobacter spp* (1 isolation), *E*. *coli* (1 isolation). Among isolates from rectum/stomia, we detected the following mechanisms of carbapenem resistance: 5 OXA-23, 4 KPC, 1 VIM. For one carbapenem resistant isolate, it was not possible to identify the mechanism of resistance since it was not among the tested ones. Xpert^®^ Carba-R detected 4 KPC, 1 VIM, 1 OXA-48. Seven out of 22 (21.8%) specimens from abdominal drainages resulted positive whereas 15 (78.2%) resulted negative for microbiological culture. Five abdominal drainage specimens resulted positive (21.7%) and 17 (78.3%) resulted negative for Xpert^®^ Carba-R. Four specimens resulted positive and fourteen resulted negative for both cultural and molecular assays. Three specimens resulted positive for microbiological culture and negative for Xpert^®^ Carba-R. One specimen resulted negative for microbiological culture and positive for Xpert^®^ Carba-R. Microbiological cultures from drainages specimens isolated the following microorganisms: *A*. *baumannii* (4 isolations), *K*. *pneumoniae* (4 isolations), *P*. *aeruginosa* (2 isolations), *Candida albicans* (1 isolation). Isolates from abdominal drainages carried the following mechanisms of resistance: 4 KPC, 2 OXA-23, 1 VIM, 1 OXA-48, 1 GES, 1 PER. Xpert^®^ Carba-R identified 4 KPC, 1 VIM, 1 OXA-48. Specimens, which tested positive by at least one method, are described in Table 2. In 9 out of 10 sets, which tested positive for carbapenem resistant bacteria in rectum/stomia specimens, a positive blood culture was also reported. When we considered positive results from drainage, in 7 out of 9 positive samples, a positive blood culture was also observed. In 5 out of 9 sets with at least one positive result by Xpert^®^ Carba-R, we observed also a positive blood culture result. Table 3 describes the inter-rater agreement of Xpert^®^ Carba-R compared to cultures from rectum/stomia and drainage specimens, along with the performance of Xpert^®^ Carba-R when we considered all genes of carbapebems resistance (both detectable and non detectable by Xpert^®^ Carba-R). The overall inter-rater agreement (Cohen’s K) for detection of carbapenem resistance for both assays was 0.47 (SE 0.14; 95% CI 0.20–0.74). The overall performance of Xpert® Carba-R for detection of carbapenem resistance was as follows: sensitivity 50% (95% CI 24.6–75.3); specificity 93.1% (95% CI 77.2–99.1); PPV 80% (95% CI 44.4–97.5); NPV 77.1% (95% 56.9–89.6). When considering the only 5 mechanisms of resistance (KPC, VIM, OXA-48, IMP-1, NDM) detected by both methods, the overall inter-rater agreement (Cohen’s K) for detection of carbapenem resistance was 0.88 (SE 0.08; 95% CI 0.71–1). The overall diagnostic performance was as follows: sensitivity was 100% (95% CI 69.1–100), specificity 94.2 (95% CI 80.8–99.3), PPV 83.3 (95% CI 59.6–97.9) and NPV 100% (95% CI 89.4–100). Table 4 summarizes the inter-rater agreement with microbiological culture and the performance of Xpert® Carba-R when considering only the 5 mechanisms of resistance (KPC, VIM, OXA-48, IMP-1, NDM) detected by both methods.

**Table 1:** Demographic and clinical characteristics of patients included in the study.

Demographic and clinical characteristics	
Age	68.5 (15.7)
(mean, SD)	
Sex	M: 14/20
	F: 6/20
Functional status	Autonomous: 12/23
	Not-autonomous 11/23
Patient origin	Surgical ward: 15/23 Operating room: 6/23
	Medical ward: 2/23
Days between hospital and ICU admission (mean, SD)	10.1 (8.67)
Duration of antibiotic therapy prior to ICU admission	5.39 (2.71)
(mean, SD)	
SOFA at the enrolment in the study (median, IQR)	(9.0, 7.25–12.0)
Type of surgery	Urgent: 22/23
	Elective: 1/23
Site of surgery	Colon (7/23; 30.4%) Colorectal (7/23; 30.4%) Small intestine (6/23; 26.1%)
	Pancreas (1/23; 4.35% Biliary tract (1/23; 4.35%) Stomach (1/23; 4.35%)
Reintervention	15/23 (65.2%)

F: female; IQR: interquartile range; M: male; SD: standard deviation. (Total number of included patients: 20. Total number of ICU admissions: 23).

**Table 2:** Descripition of positive specimens from at least one site by either microbiological cultures or Xpert Carba-R. Genetic determinants (GDs) of carbapenem resistance were detected by in-house PCR.

Rectum/stomia culture	GDs	Rectum/stomia Xpert Carba-R	Drainage culture	GDs	Drainage Xpert Carba-R	Blood culture
*P*. *aeruginosa*	*GES+ PER+*	Negative	Negative	*/*	Negative	*P*. *aeruginosa*
Negative	*/*	Negative	*A*. *baumannii*	*OXA-23*	Negative	Negative
*K*. *pnumoniae Enterobacter spp*.	*KPC VIM OXA-23*	*KPC*	*K*. *pneumonia Enterobacter spp*.	*KPC VIM OXA-23*	*KPC VIM*	*K*. *pneumonia A*. *baumannii*
		*VIM*				
			*A*. *baumannii*			
*A*. *baumannii*						
*K*. *pneumoniae*	*KPC*	*KPC*	*K*. *pneumoniae*	*KPC*	*KPC*	*K*. *pneumoniae*
*A*. *baumannii*	*OXA-23*	Negative	*A*. *baumannii*	*OXA-23*	Negative	*A*. *baumannii*
Negative	*/*	Negative	*P*. *aeruginosa*	*GES+PER+*	Negative	Negative
*K*. *pneumoniae*	*KPC*	*KPC*	*K*. *pneumoniae*	*KPC*	*KPC*	*K*. *pneumoniae*
*A*. *baumanniii*	*OXA-23*	Negative	*A*. *baumannii*	*OXA-23*	Negative	*A*. *baumannii*
*P*. *aeruginosa*	*Not detected*	Negative	Negative	*/*	Negative	Negative
*A*. *baumannii*	*OXA-23*	Negative	Negative	*/*	Negative	*A*. *baumanii*
*K*. *pneumoniae*	*KPC*	*KPC*	*K*. *pneumoniae*	*KPC*	*KPC*	*K*. *pneumoniae*
Negative	*/*	*OXA-48*	Not available	*/*	Not available	Negative
*A*. *baumannii K*. *pneumoniae*	*OXA-23 KPC*	*KPC*	*A*. *baumannii*	*OXA-23 KPC*	*KPC*	*A*. *baumanii*
			*K*. *pneumoniae*			
Negative	*/*	Negative	Negative	*/*	*OXA-48*	Negative

GDs = genetic determinants of carbapenem resistance

**Table 3:** Performance of Xpert Carba-R in comparison with microbiological culture for detection of carbapenem resistance by all genes (detectable and non detectable by both methods) in our cohort of patients.

	Rectum/stomia specimen	Drainage specimen	Overall
Inter-rater agreement (Cohen’s K)	0.41 (SE 0.19; 95% CI 0.04–0.77)	0.44 (SE 0.19; 95% CI 0.08–0.82)	0.47 (SE 0.14; 95% CI 0.20–0.74)
Sensitivity	44.4% (95% CI 13.7–78.8)	57.1% (95% CI 18.4–90.1%)	50% (95% CI 24.6–75.3)
Specificity	92.9% (95% CI 66.1–99.8);	93.3% (95% CI 68.1–99.8)	93.1% (95% CI 77.2–99.1)
PPV	80% (95% CI 28.4%–99.5%);	80% (95% CI 28.4–99.5%)	80% (95% CI 44.4–97.5)
NPV	72.2% (95% CI 46.5–90.3)	82.4% (95% 56.6–96.2)	77.1% (95% 56.9–89.6)

PPV = positive predictive value; NPV = negative predictive value

**Table 4:** Performance of Xpert Carba-R assay in comparison with microbiological culture for detection of carbapenem resistance when considering only the 5 mechanisms of resistance (KPC, VIM, OXA-48, IMP-1, NDM) detected by both methods.

	Rectum/stomia specimen	Drainage specimen	Overall
Inter-rater agreement (Cohen’s K)	0.88 (SE 0.11, 95% CI 0.65–1)	0.87 (SE 0.11, 95% CI 0.64–1)	0.88 (SE 0.08, 95% CI 0.71–1)
Sensitivity	100% (95% CI 47.8–100)	100% (95% CI 47.8–100%)	100% (95% CI 69.1–100)
Specificity	94.4 (95% CI 72.7–99.8)	94.1 (95% CI 71.3–99.8)	94.2 (95% CI 80.8–99.3)
PPV	83.3 (95% CI 35.8–99.5)	83.3 (95% CI 35.8–99.6)	83.3 (95% CI 59.6–97.9)
NPV	100% (95% CI 80.5–100)	100 (95% CI 79.4–100)	100% (95% CI 89.4–100)

SE = standard error, PPV = positive predictive value; NPV = negative predictive value

## Discussion

To our knowledge, this is the first study specifically testing the clinical diagnostic usefulness of Xpert^®^ Carba-R assay in a cohort of septic abdominal patients.

In all specimens resistant to carbapenems by phenotypical method (E-test) and possessing genes detectable by Xpert® Carba-R, there was a concordance between the two methods.

In some cases, we registered carbapenem resistance to culture-based method along with negative Xpert^®^ Carba-R. In 6 of these cases, in-house PCR detected OXA-23 and, in other 2 cases, GES and PER. In another case, the gene responsible for carbapenem resistance was not identified since all in-house PCR resulted negative. According to our results, we may argue that the inclusion of additional carbapenem resistance genes in the molecular tests (e.g. *OXA-23*) would improve the diagnostic performance and clinical usefulness of this technique. In two patients samples, Xpert^®^ Carba-R identified *bla*_*OXA-48*_ as responsible mechanism of carbapenem resistance. In these 2 samples, a late and weak positive signal was observed and no carbapenem resistant strain was isolated on McConkey Agar plate. We supposed that these two results were possibly related to non-vital bacteria or residual DNA or that the amount of bacterial cells in the clinical specimen was below the detection limit of the culture-based method. This discrepancy in isolation is common among PCR techniques applied to microbiological diagnosis, leading to unclear clinical significance. [11] Discrepancies between molecular and standard methods are well known for OXA-48 gene family because of its low enzymatic activity against carbapenems yielding MICs lower than the resistance cut-off value. Nevertheless, OXA-48-like-producing bacteria are typically resistant to carbapenems, as a consequence of concurrent resistance mechanisms, such as porin defects or production of other β-lactamases. [22] In our study, mean turnaround time for results by Xpert^®^ Carba-R was less than one hour and half, compared to about 3 days required by culture-based method. Although this difference may be an advantage in terms of timing of effective antibiotic therapy institution, clinicians should be aware of the limitations of the technique.

To date, Xpert^®^ Carba-R diagnostic performance has been studied in comparison with standard blood cultures and other PCR techniques. A recent report from Saudi Arabia described the failure of Xpert^®^ Carba-R to detect multiple coexisting resistance mechanisms. [23] Conversely, in one of our cases, Xpert^®^ Carba-R was able to detect two coexisting mechanisms of resistance (KPC and VIM). A report from Korea studied the performance of Xpert^®^ Carba-R, in comparison with standard culture methods, in an unselected ICU cohort of patients to determine intestinal colonization rates of carbapenemase-producing organisms. Clinical samples included stool samples and rectal swabs. Of note, duplicates samples from the same patients were excluded. Moreover, authors did not calculate an overall measure of concordance between standard cultures and Xpert^®^ Carba-R. [14] More recently, reports of inability to detect OXA-48-like genes have been published. *OXA-181* is an allele of the OXA-48 family. More than 120 alleles of the so-called *big 5 genes* of carbapenem resistance genes have been identified, leading to a potential reduced sensitivity of the RT-PCR assay. Notably, *OXA-48* was subsequently added to the original 4-gene detection kit of Xpert^®^ Carba-R in order to cover the big five genes most frequently responsible for carbapenem resistance according to different epidemiologic reports. [24] Due to the emergence and spread of different genes of resistance other than *the big five* (e.g. *OXA-23*) and alleles within each family of genes of carbapenem resistance (e.g. *OXA-181*), [25] Xpert^®^ Carba-R and other RT-PCR based techniques should be considered a dynamic compromise for detection of currently most influent genes.

This study has some limitations. Firstly, our convenience sample size was relatively small due to our limited available resources for this study. However, to our knowledge, it was the first attempt to study the performance of the Xpert^®^ Carba-R assay in this setting before a more extended use. Another limitation may be attributed to the limited number of genes detected by the Xpert^®^ Carba-R. According to a review of our microbiological reports of the last 3 years, *bla*_KPC_, *bla*_NDM_, *bla*_VIM_, *bla*_OXA-48_, and *bla*_IMP-1_ accounted for the majority of genes responsible for carbapenem resistance in our ICU. However, this could not prevent the emergence of other carbapenem resistance mechanisms during the study period. Indeed, in 3 cases we detected OXA-23 whereas GES and PER in two cases. In one occasion, the mechanism responsible for carbapenem resistance was not identified, since it was not among the tested PCRs. We may argue that the inclusion of additional resistance genes in the molecular panel would improve the clinical usefulness of this technique. However, the spreading of different resistance genes underlines the limitation of molecular methods and the need to realize and use specific panels of molecular tests according to local epidemiological data. Clinicians should be aware of the most commonly mechanisms of resistance, related to local epidemiology and specific features of each ward as well, before introducing any molecular method in their daily practice and evaluate its performance. Anyway, standard culture should be always performed together with molecular tests, not only to identify those bacteria harbouring other resistance mechanisms, but also to isolate the strain for subsequent analyses. We believe that additional studies should be performed to assess validity of this technique especially as a supportive tool for decision about timely antimicrobial strategy.

## Conclusions

Xpert^®^ Carba-R may be considered an additional diagnostic tool for early detection of carbapenem resistance among critically ill patients with abdominal sepsis. Local epidemiology strongly influences the proportion of multidrug-resistant infected patients detectable by this molecular method. Clinicians should be aware of local microbiological epidemiology before introducing Xpert^®^ Carba-R in the diagnostic protocol of their ICUs. The inclusion of additional genes of carbapenem resistance in the panel of Xpert^®^ Carba-R may improve its diagnostic performance and clinical usefulness.
